# Should Subclinical Hypothyroidism Be an Exclusion Criterion for the Diagnosis of Polycystic Ovary Syndrome?

**Published:** 2017

**Authors:** Sebastião Freitas de-Medeiros, Márcia Marly Winck Yamamoto, Matheus Antônio Souto de-Medeiros, Jacklyne Silva Barbosa, Robert John Norman

**Affiliations:** 1- Federal University of Mato Grosso, Cuiabá, Mato Grosso, Brazil; 2- Tropical Institute of Reproductive Medicine and Menopause, Cuiabá, Mato Grosso, Brazil; 3- University of Adelaide, Robinson Research Institute and Fertility SA, Adelaide, Australia

**Keywords:** Hyperandrogenism, Hypothyroidism, Polycystic ovary syndrome, Thyroid hormones

## Abstract

**Background:**

The purpose of the study was to examine whether patients with subclinical hypothyroidism (SCH) should be excluded before making a diagnosis of polycystic ovary syndrome (PCOS).

**Methods:**

Seven hundred sixteen patients, 462 with true PCOS, 31 with PCOS-SCH, and 223 normal cycling women were enrolled. Clinical, metabolic, and hormonal parameters among the groups were investigated. Continuous variables were compared by one-way analysis of variance. Proportions were compared using Z test. Fisher test was used to compare categorical variables. Simple correlation was performed using Spearman’s coefficient. Correlation between thyroid stimulating hormone (TSH) and dependent variables were performed using backward multiple regression. The significance level was set at 0.05.

**Results:**

True polycystic ovary and polycystic ovary with subclinical hypothyroidism patients presented similar anthropometrical parameters. C-peptide was higher in polycystic ovary patients than in the other groups (p=0.014). Prevalence of glucose intolerance (p=0.186) and insulin resistance (p=0.293) was not statistically different in polycystic ovary and polycystic ovary with subclinical hypothyroidism. TSH levels showed positive correlation with lean body mass (p=0.032), total cholesterol (p=0.046, insulin (p=0.048) and prolactin (p=0.047). Backward multiple regression model retained TC, insulin, and PRL as predictors of TSH levels (p=0.011).

**Conclusion:**

Anthropometric parameters and ovary morphology were similar in both PCOS and PCOS-with-SCH patients. Regarding hormones, only C-peptide was higher in PCOS group. TSH correlated with total cholesterol, insulin, and prolactin. Before PCOS diagnosis, the exclusion criterion thyroid dysfunction should be standardized and subclinical hypothyroidism should not exclude a diagnosis of PCOS.

## Introduction

In women of reproductive age, depending on the diagnostic criteria used, the prevalence of polycystic ovary syndrome (PCOS) ranges from 5 to 21% (1, 2). The condition is characterized by oligo/anovulation, hyperandrogenism, and polycystic ovary morphology (3). Dyslipidemia, dysglycemia, and insulin resistance are frequent metabolic abnormalities, increasing risks of type II diabetes mellitus (T2DM) and cardiovascular disease (CVD) (4). Primary hypothyroidism patients share many signs and symptoms with PCOS, such as menstrual disorders, infertility, hyperandrogenism, and weight gain (5, 6). Among the endocrine features, patients with primary overt hypothyroidism may have mild increase in total testosterone (T) and free testosterone (fT) total and free estradiol, prolactin (PRL) and luteinizing hormone (LH), and decreased sex hormone binding globulin (SHBG) levels (6, 7). Dyslipidemia and insulin resistance are frequent metabolic abnormalities with increased risk of type II diabetes mellitus (T2DM) and cardiosvascular diseases (CVD) (8). Moreover, ovaries with bilateral multicystic appearance are frequently found in these patients (9).

So, called subclinical hypothyroidism (SCH), found between 3–8% of women of reproductive age (10), has few signs or symptoms of thyroid dysfunction and often remains untreated; whether SCH leads to clinical, endocrine, or metabolic alterations that could be misdiagnosed as the early stages of PCOS remains to be demonstrated. Nearly 14% of SCH patients may present dyslipidemia, dysglycemia, insulin resistance, infertility, ovulatory dysfunction, obesity, and abnormal menstrual cycle, mimicking PCOS (5, 7). High levels of total (T) and free testosterone (fT), LH, PRL, fasting and postprandial insulin, glycated hemoglobin (HbA1C), homeostasis model assessment of insulin resistance index (HOMA-IR), serum lipoprotein (a), triglyceride, total cholesterol (TC), unfavorable low-density lipoprotein (LDL) and high-density lipoprotein (HDL) and low levels of SHBG (8, 11), are independent risk factors for metabolic syndrome or myocardial infarction frequently described in SCH (11–13).

Polycystic ovary syndrome patients with SCH patients (PCOS-with-SCH) may be just like those with true PCOS and the SCH is incidental. It has been proposed that exclusion of hypo- or hyperthyroidism is not mandatory to make a diagnosis of PCOS in the absence of other symptoms or signs of thyroid dysfunction (14). However, according to the current recommendation, definitive PCOS diagnosis should be made after exclusion of thyroid dysfunction (3, 14). This has not been universally accepted however (7, 14, 15) and, in many publications, TSH levels are sometimes not measured or are dismissed in brief statements excluding thyroid function (16). Criteria frequently used to exclude thyroid disorders nowadays include overt hypothyroidism (14), clinical suspicion of hypothyroidism (17), and different levels of TSH (18–21). Given the lack of a clear guideline on this matter and the required standardization among researchers, the present study aimed to examine whether patients with subclinical hypothyroidism, defined as TSH concentrations ≥4.2 to ≤10 *μUI/ml* with normal FT4, should be excluded from patients with initial diagnosis of PCOS. To assess this possibility, comparisons of clinical, endocrine, and metabolic features among PCOS, PCOS-with-SCH patients, and normal ovulatory women were performed.

## Methods

This research was conducted at a University Hospital and Tertiary Reproduction Unit and approved by the local Committee for Ethics at The Julio Muller University Hospital (Approval decision of Ethic Committee number: 093/FCM/03-Federal University of Mato Grosso, Cuiabá, MT, Brazil). Each patient signed an informed consent. PCOS patients, PCOS-with-SCH patients (TSH level <10 *μUI/ml*), and non-PCOS normal ovulatory patients with TSH levels <4.2 *μUI/ml* were prospectively enrolled, from March 2003 until September 2013 at the Fertility Clinics of The Julio Muller University Hospital and Tropical Institute of Reproductive Medicine and Menopause in Cuiabá, Brazil. Using accessibility sampling, patients were included at first visit according to eligibility criteria requirement. None of the women included as controls had thyroid dysfunction, a history of infrequent menses or amenorrhea or clinical sign of hyperandrogenism. A total of 716 women were included in the present study: 461 PCOS with normal thyroid function, 31 PCOS-with-SCH, and 223 normal controls. PCOS patients were diagnosed using the Rotterdam criteria (3). Patients who have used sex steroids or insulin-sensitizing drugs in the previous 6 months and patients with overt hypothyroidism were excluded.

Primary hypothyroidism was defined by a TSH level ≥4.2 *μUI/ml* and FT4 levels ≤0,7 *ng/dl* (9 *pmol/l*) (22). True PCOS was defined using Rotterdam criteria and those patients with diagnostic features of PCOS but with a TSH level between 4.2 *μUI/ml* and ≤10 *μUI/ml* were defined as PCOS-with-SCH patients. Normal ovulatory women, most of them diagnosed as infertile because of non-ovarian causes, were included as controls. All patients underwent intensive examination for the presence of oligomenorrhea, amenorrhea, and signs of hyperandrogenism (acne, hirsutism, alopecia) or clinical features of insulin resistance (acanthosis nigricans and acrochorda). Systolic (SBP) and diastolic blood pressure (DBP), and anthropometric parameters were measured. The subjects were weighed on an electronic scale, and their height was measured using a Harpenden stadiometer (Holtain Limited, Crymych, Dyfed, UK). The waist was measured at the midway point between the lower rib margin and the iliac crest, and the hip was measured from the widest circumference over the great trochanters. The body mass index (BMI) was calculated as body weight (*kg*)/height (meter) squared. Obesity was defined as a body mass index (BMI) ≥30 *kg/m*^2^. Lean body mass (LBM) was calculated according to James’ formula: (1.07 ×weight *kg*)–148 (weight^2^/100×height *m*^2^) (23). Fat mass (FM) was calculated as body weight minus LBM. Abdominal adiposity was estimated by the conicity index (CI), using the following equation: $[(\text{WC}(\text{m})/\sqrt{\text{BW}(\mathrm{kg}):\text{height}(\text{m})]}$ (24).

Blood samples were obtained between 7:00 and 9:00 am by venupuncture after a 10–12 *hr* fast. All patients with regular cycles were tested in the early follicular phase of the menstrual cycle (days 3–5 of the cycle). Patients with oligomenorrhea or amenorrhea had their blood collected at any time provided the progesterone was less than 2 *ng/ml* (6.4 *nmol/L*). Triglycerides (TG), high-density lipoprotein cholesterol (HDL-C), and total cholesterol (TC) levels were measured using an enzymatic assay (Wiener Laboratories, Rosário, Argentina). Low-density lipoprotein cholesterol (LDL-C) was calculated as TC-(HDL-C+TG/5) (25). Glucose concentration was analyzed using the glucose oxidase technique (Beckman Glucose Analyses, Fullerton, CA, USA). Subjects were diagnosed with impaired fasting glucose (IFG) when fasting plasma glucose (FPG) level was between 100 *mg/dL* (5.5 *mmol/L*) and 126 *mg/dL* (6.99 *mmol/L*) (26), and in 2 *hr* oral glucose tolerance test (GTT), glucose level was between 140 *mg/dL* (7.8 *nmol/L*) and 199 *mg/dL* (11.0 *nmol/l*) (26). Implied insulin resistance (IR) was defined using fasting insulin levels >12.2 *μU/ml* (84.7 *pmol/L*) (27) and/or HOMA-IR ≥2.8 (28). HOMA%β was estimated using the following equation: HOMA% B=[(20×fasting insulin *μUI/ml*)/fasting glucose (*nmol/L*) – 3.5] (28). For the GTT, blood samples were collected at 0, 30, 60, 90, 120, and 180 *min* after ingestion of 75 *g* of dextrose for the measurement of plasma glucose and insulin levels (29).

Hormone levels were measured using previously validated methods and their imprecisions in the local laboratory were extensively presented in previous publications (29). High-performance liquid chromatography (HPLC)/radioimmunoassay (RIA) was performed for compound S (11-desoxy-cortisol) measurement following extraction using a method developed in-house by the Alvaro Center of Analysis and Clinical Research in Paraná, Brazil. 17-hydroxypregnenolone level was measured with an HPLC/RIA using titrated steroids (NEN Life Science Products, Boston, MA, USA) and antiserum from ICN Biochemical Inc (Costa Mesa, CA, USA). The free androgen index (FAI) was estimated as the total T (*nmol/L*)/sex hormone-binding globulin (SHBG), *nmol/L*×100. Taking into account the limited agreement with respect to the Ferriman–Gallwey score, a specific value to define hirsutism was not used and clinical hyperandrogenism was defined as a binomial variable by the single presence or absence of hirsutism (30). TSH and free thyroxine were measured using electrochemiluminescence assays (Elecsys 1010, Roche Diagnostics GMBH, Mannhein, Germany). Ovary transvaginal ultrasonography was performed using a Voluson machine (Voluson® E8, GE Health Care, England) and PCO morphology was defined according to previous recommendations (31).

### Statistical analyses:

Data were missing in some cases because of unavailable vital signs, inadequate specimens, or subjects not providing specimens. Anthropometrical, biochemical, and endocrinological continuous data are presented as mean and standard deviation (SD) and compared using one-way analysis of variance and the Tukey post hoc test. The Z test was used to compare two categorical variables. Fisher exact test was used to compare more than two categorical variables. Correlations between TSH and other variables were performed using Spearman’s rho correlation coefficient. Further, using TSH as the criterion variable, backward regression analysis including variables that presented a significant simple correlation coefficient with TSH was performed to determine the best model that could be used to examine the weight of different predictors in the criterion variable. Durbin-Watson test was used to verify correlation between residuals. All tests were two-sided, and the significance level was set at 0.05. All analyses were performed using SPSS for Windows, version 17 (SPSS Inc., Chicago, IL, USA).

## Results

Patients with true PCOS were younger than those in the control group (p=0.010), but importantly, as the primary outcome of the study, PCOS and PCOS-with-SCH subjects presented similar ages (p=0.088). The number of patients with acne was higher in PCOS with SCH than in PCOS patients (42.08% *vs*. 51.61%, p=0.001). On the other hand, hirsutism (50.54% *vs*. 38.7%) was higher in PCOS than in PCOs-with-SCH patients (p<0.001). Acanthosis nigricans and PCOS morphology were of similar prevalences in PCOS and PCOS-with-SCH (p=0.468 and p=0.48, respectively) but these conditions were more frequent in PCOS and PCOS-with-SCH than in controls (p<0.001 and <0.001, respectively). Clinical and anthropometrical characteristics among the three groups of patients are compared in table 1. All anthropometric variables were equal in PCOS and PCOS-with-SCH and higher in control subjects (p<0.001). Weight, waist circumference, W:H ratio, CI, and LBM were higher in PCOS-with SCH than in controls (p<0.001). As depicted in table 2, all metabolic markers were significantly different when PCOS patients were compared with controls. PCOS-with-SCH patients had higher fasting glucose, glucose 120 and total cholesterol levels and HOMA%β than controls as well as lower HDL-C. After comparing PCOS with PCOS- with-SCH, only C-peptide levels showed to be higher in PCOS.

**Table 1.** Comparison of clinical and anthropometrical characteristics among PCOS and PCOS-with-SCH patients, and normal controls ^*^

Variable**	**PCOS TSH <4.2 *μUI/ml***		**PCOS-with-SCH TSH ≥4.2 *μUI/ml***		**Control TSH <4.2 *μUI/ml***	

	**n**	**Mean±SD**	**n**	**Mean±SD**	**n**	**Mean±SD**
**Age (years)**	460	26.72±5.38	30	28.73±5.21	223	30.34±4.74 ^a^
**SBP (*mmHg*)**	407	118.82±12.43	27	119.63±15.80	206	115.11±7.87 ^a^
**DBP (*mmHg*)**	407	77.13±9.37	27	75.97±8.36	206	73.82±8.11 ^a^
**Weight (*kg*)**	443	74.34±17.51	31	75.17±19.73	215	63.95±10.84 ^a,b^
**BMI (*kg/m^2^*)**	412	29.11±6.74	26	26.75±6.18	206	24.47±4.03 ^a^
**Waist (*cm*)**	385	86.02±15.29	25	83.72±15.17	204	73.93±9.39 ^a,b^
**Hip (*cm*)**	384	106.68±12.36	25	105.68±12.57	204	100.12±8.58 ^a^
**W:H ratio**	384	0.80±0.08	25	0.79±0.07	204	0.73±0.06 ^a,b^
**C I**	378	1.15±0.10	23	1.14±0.09	203	1.07±0.07 ^a,b^
**LBM (*kg*)**	412	45.61±6.37	25	44.85±5.91	216	20.67±7.17 ^a,b^
**FM (*kg*)**	412	28.76±11.99	26	25.21±11.45	201	20.66±6.86 ^a^

SBP, systolic blood pressure; DBP, diastolic blood pressure; BM, body mass index; CI, conicity index; LBM, lean body mass; FM, fat mass

* One way analysis of variance followed by post hoc Tukey test;

** PCOS=patients with PCOS and TSH <4.2 *μUI/ml*.

PCOS-with-SCH=patients with PCOS and TSH >4.2 *μUI/ml*. Control=normal women with TSH <4.2 *μUI/ml*

a: p<0.001 PCOS *vs*. control;

b: p<0.001 PCOS-with-SCH *vs*. control

**Table 2.** Comparison of metabolic features among PCOS, PCOS-with-SCH and controls ^*^

Variable **	**PCOS TSH <4.2 *μUI/ml***		**PCOS-with-SCH TSH ≥4.2 *μUI/ml***		**Control TSH <4.2 *μUI/ml***	

	**n**	**Mean±SD**	**n**	**Mean±SD**	**n**	**Mean±SD**
**Fasting glucose (*mmol/l*)**	422	5.06±0.61	30	5.06±0.45	195	4.70±0.38 ^a,b^
**Fasting insulin (*μmol/l*)**	388	97.36±63.96	23	77.75±46.90	136	52.60±30.00 ^a^
**Glucose 120 (*mmol/l*)**	274	6.71±2.17	17	6.09±1.66	94	5.24±1.43 ^a,b^
**HbA1C (*%*)**	305	5.76±1.22	22	5.40±0.99	152	4.98±0.59 ^a^
**HOMA-IR**	378	1.80±1.15	22	1.48±0.85	129	0.96±0.54 ^a^
**HOMA%B**	379	134.35±56.60	23	152.02±131.01	129	104.06±38.88 ^a,b^
**C-peptide (*nmol/l*)**	277	0.81±0.38	17	0.58±0.27	131	0.49±0.23 ^a,c^
**TC (*mmol/l*)**	380	4.80±2.23	25	4.58±0.92	177	4.36±1.39 ^a,b^
**HDL-C (*mmol/l*)**	371	1.17±0.28	24	1.14±0.27	176	1.36±0.30 ^a,b^
**LDL-C (*mmol/l*)**	369	2.86±0.79	25	2.84±0.78	174	2.56±0.66 ^a^
**TG (*mmol/l*)**	379	1.41±0.90	25	1.47±0.63	177	1.11±0.79 ^a^

HbA1C, glycated hemoglobin; HOMA, homeostasis model assessment; TC, total cholesterol; HDL-C, high density lipoprotein - cholesterol; LDL-C, low density lipoprotein-cholesterol; TG, triglyceride

* One way analysis of variance followed by post hoc Tukey test;

** PCOS= patients with PCOS and TSH<4.2 *μUI/ml*.

PCOS-with-SCH = patients with PCOS and TSH >4.2 *μUI/ml*. Control = normal women with TSH <4.2 *μUI/ml*

a: p<0.001 PCOS *vs*. control;

b: p<0.001 PCOS-with-SCH *vs*. control;

c: p=0.014 PCOS *vs*. PCOS-with-SCH

Using IFG as a marker, glucose intolerance was more prevalent in PCOS (13.9%) and PCOS-with-SCH groups (16.1%) than in control group (1.24%) (p=0.001) and not statistically different between PCOS and PCOS-with-SCH (p=0.726). Using glucose levels at GTT 120 *min* time point, glucose intolerance was more prevalent in PCOS (10.6%) than in controls (2.5%) (p<0.001) but there was not any significant difference between PCOS (10.6%) and PCOS-with-SCH patients (3.2%) (p=0.186). Hyperinsulinemia, taken as fasting insulin ≥12.2 *μUI/ml* (84.73 *pmol/L*), was also more frequent in PCOS (41.8%), and PCOS-with-SCH patients (32.2%) than in controls (10.4%) (p<0.001); PCOS and PCOS-with-SCH had similar prevalences of imputed insulin resistance (p=0.293). Using a cutoff HOMA-IR of 2.8, 15.83% of PCOS patients and 9.7% of PCOS-with-SCH had higher HOMA-IR than controls (0.8%) (p<0.001). HOMA-IR was not statistically different in PCOS and PCOS-with-SCH patients.

Regarding endocrine parameters, only C-peptide (Table 2), TSH and FT_4_ levels were different between PCOS and PCOS-with-SCH patients (Table 3). Interestingly, PRL was lower in PCOS than in controls (p<0.001). Sex steroids, LH and FAI were equally higher in PCOS and PCOS-with-SCH than in controls. TSH and FT4 concentrations were similar between PCOS and controls. FT4 concentrations were lower in PCOS-with-SCH patients compared with PCOS and control patients but yet in the normal range. TSH levels were associated with fasting glucose (rho=0.170; p=0.016) but were not associated with any anthropometric, or endocrine parameters in the control group. In PCOS patients, TSH levels had a weak positive correlation with LBM (rho=0.110; p=0.032), TC (rho=0.105; p=0.046), insulin (rho= 0.103; p=0.048) and PRL (r=0.103, p=0.047). In PCOS-with-SCH patients, TSH levels presented positive correlation only with TC (rho=0.430; p= 0.028) and HDL-C concentrations (rho=0.432, p= 0.031). The importance of these associations was further tested using a backward multiple regression. Considering the model (Table 4), the variability of TSH accounted for by all predictors was only 2.8% (R^2^=0.028) and this model is a good fit of the data (F=3.771, p=0.011). The exclusion of LBM from the model decreased the TSH variability in 1.6% (0.044–0.028). The contribution, given by standardized coefficients, of each predictor variable was as follows: TC (B=0.113, t=1.919, p=0.056), insulin (B=0.136, t=2,291, p=0.023), and PRL (B=0.107, t=1.799, p=0.073).

**Table 3.** Comparison of endocrinological characteristics among PCOS, PCOS-with- SCH patients and controls ^*^

Variable **	**PCOS TSH <4.2 *μUI/ml***		**PCOS-with-SCH TSH ≥4.2 *μUI/ml***		**Control TSH <4.2 *μUI/ml***	

	**n**	**Mean±SD**	**n**	**Mean±SD**	**n**	**Mean±SD**
**TSH (*mmol/l*)**	417	1.99±0.86	30	7.83±7.48	195	1.96±0.81^a,b^
**FT4 (*pmol/l*)**	402	14.31±2.39	27	12.68±2.91	191	13.93±2.08 ^a,c^
**PRL (*nmol/l*)**	404	558.02±293.24	27	586.80±237.98	182	627.69±287.7^d^
**E2 (*pmol/l*)**	301	185.57±66.67	18	173.08±58.46	157	163.93±63.48 ^e^
**Tt (*nmol/l*)**	397	2.03±0.92	26	1.69±0.63	155	1.03±0.48 ^b,e^
**FT (*nmol/l*)**	370	0.05±0.03	26	0.04±0.04	151	0.01±0.009 ^b,e^
**SHBG (*nmol/l*)**	363	35.58±19.88	23	37.73±21.17	145	64.84±32.71^b,e^
**FAI (*%*)**	352	7.46±5.37	21	5.97±3.70	139	2.05±1.85 ^b,e^
**FSH (*mUI/ml*)**	397	5.77±1.77	25	6.43±1.61	178	7.18±2.14^e^
**LH (*mUI/ml*)**	394	10.06±5.80	27	8.39±3.94	175	5.08±2.59 ^b,e^
**17-OHP4 (*nmol/l*)**	390	0.038±0.021	27	0.033±0.014	145	0.028±0.030 ^e^
**DHEAS (*nmol/l*)**	383	4.92±2.37	24	4.42±1.60	153	3.91±1.96 ^e^
**Androstenedione (*nmol/l*)**	378	0.110±0.111	26	0.12±0.18	144	0.075±0.215^d^

* One way analysis of variance followed by post hoc Tukey test;

** PCOS=patients with PCOS and TSH<4.2 *μUI/ml*.

PCOS-with-SCH=patients with PCOS and TSH>4.2 μUI/ml. Control=normal women with TSH <4.2 *μUI/ml*

a: p<0.001 PCOS *vs*. PCOS-with-SCH;

b: p<0.001 PCOS-with-SCH *vs*. control;

c: p=0.041 PCOS-with-SCH *vs*. control;

d: p=0.007 PCOS *vs*. Control;

e: p<0.001 PCOS *vs*. control

**Table 4.** Backward multiple regression between TSH and significant predictors found after simple linear correlation

**Model a**	**R**	**R square**	**Adjusted R square**	**Std. error of the estimate**	**F**	**Durbin-Watson**	**p-value**
**1**	0.211^b^	0.044	0.031	0.85480	3.289	--	0.012^b^
**2**	0.196^c^	0.038	0.028	0.85602	3.771	1.971	0.011^c^

a: Criterion variable: TSH *mUI/ml*;

b: Predictors (constant), PRL, LBM, TC, insulin;

c: Predictors (constant), PRL, TC, insulin

## Discussion

All current diagnostic criteria of PCOS emphasize the importance of excluding thyroid dysfunction for accepting a definitive diagnosis, suggesting that a high TSH level in the presence of normal thyroxin concentration (so-called subclinical hypothyroidism) should be an exclusion criterion from a precise diagnosis of PCOS. The present study was designed to examine this group of patients and to compare them with definitive PCOS and definite control subjects.

In summary, the current study demonstrated higher blood pressure in PCOS patients than in controls. Anthropometrical features were similar in PCOS and PCOS-like-SCH in most of the patients. Body weight, waist circumference, W-H ratio, CI, and LBM were higher in PCOS-like-SCH than in controls. These two groups presented similar BMI, hip circumference, and fat mass. Even though PCOS patients presented different metabolic profile than controls, PCOS-like-SCH and controls had similar concentrations of fasting insulin, HbA1C, HOMA-IR, C-peptide, LDL-C and TG. Only C-peptide levels were significantly higher in PCOS than in PCOS-like-SCH patients. Free thyroxin was lower in PCOS-like-SCH than in PCOS and control patients. Estradiol levels were higher in PCOS and PCOS-like-SCH patients than in control group. PCOS and PCOS-like-SCH patients presented similar concentrations of LH, androgens and FAI. TSH levels presented weak positive correlation with LBM, TC, insulin, and PRL, in PCOS patients. In the PCOS-like-SCH group, TSH was positively correlated only with TC and HDL-C concentrations.

A few possible limitations need to be considered when interpreting the present findings. The normal upper limit of the TSH level is not standardized yet. The adopted cutoff for TSH level of 4.2 *μUI/ml* in the current study was higher than that proposed by several authors (7, 18–20, 32, 33) and lower than those used in other studies (21, 32). The small sample size in PCOS-like-SCH group may have reduced the power of the comparisons. On the other hand, the current findings have scientific strength. One of the strengths of the study is the large number of patients examined prospectively with comprehensive anthropometric, metabolic and endocrine investigations. Additional in-sights into the relevance of establishing a single cutoff for TSH level in PCOS patients’ diagnosis are provided, and the results of the present study should be considered for the exclusion of thyroid disorders before the diagnosis of true PCOS.

The finding of an equal prevalence of many signs of hyperandrogenism and PCOS morphology, in PCOS and PCOS-like-SCH patients demonstrated the high clinical similarity between these two conditions and confirms previous observations (7, 19, 32–35). Although a clear diagnosis of PCOS-like-SCH had been established in 43% of patients from a group of patients presenting with PCOS and autoimmune thyroiditis in a previous study, the entire group also had equal prevalence of acne, hirsutism, androgenic alopecia, and PCO morphology (35). Another study found the same degree of hirsutism, as evaluated by Ferriman-Gallwey score, in PCOS and PCOS-like-SCH patients (32). Even some studies had shown that clinical hyperandrogenism is not common in patients with hypothyroidism (14, 17), biochemical increase in androgen levels, mainly in total, free testosterone and FAI in hypothyroidism are frequently found (18, 34, 36).

Anthropometrical parameters were equal in PCOS and PCOS-like-SCH patients in the current study. Similar BMI and WHR in PCOS and PCOS-like-SCH were already reported in several studies (19–21, 32, 34, 37). Increased BMI was found in a study when PCOS patients with TSH >2 *μUI/ml* and PCOS patients with lower TSH levels were compared (7). Regarding carbohydrate metabolism, only C-peptide was higher in PCOS than in PCOS-like-SCH. Supporting the current study, the PCOS-with-SCH subjects were equal to those of PCOS with respect to fasting glucose, glucose after GTT_120_, and HOMA-IR was also found in other studies (9, 21, 32, 34, 37). Higher or equal fasting insulin between PCOS and PCOS-like-SCH patients were also previously reported (18, 19, 32). Notably, increased HOMA-IR in PCOS-like-SCH patients was reported only in one study (7).

In general, lipid levels are similar between PCOS and PCOS-like-SCH (18, 37). TG levels have been found to be lower (37), higher (18, 21) or similar (34) in PCOS-like-SCH patients when compared with PCOS patients. Although HDL-C levels had been equal in PCOS and PCOS-like-SCH patients in the current study, other studies have shown lower levels of this lipid in PCOS-like-SCH patients (18, 34). Higher TC levels were also seen in PCOS-like-SCH patients in other studies (18, 34) LDL-C level was reported to be higher in PCOS-like-SCH patients, in addition to showing a positive correlation with TSH levels (21, 34). No difference in lipid levels between SCH patients and controls was reported in non-PCOS populations (38), but in the current study, HDL-C levels were lower in PCOS-with-SCH than in controls.

With the exception of C-peptides, in the present study, PCOS and PCOS-like-SCH patients showed to be endocrinologically equal. Results of other studies comparing hormonal parameters between PCOS and PCOS-like-SCH have not been consistent. Higher levels of T, fT, and FAI in PCOS than in PCOS-like-SCH were found in a few reports (7, 19). On the other hand, other studies reported equal concentrations of these hormones in both groups (32, 34). Higher levels of DHEAS, PRL, and LH in PCOS-like-SCH compared with PCOS were also reported (32, 39), but equal concentrations of LH, FSH, T, PRL, DHEAS and E2 were also found by others (7, 19, 21, 34). Lower levels of SHBG in PCOS-like-SCH patients than in PCOS patients were reported in only one study (18). Free thyroxine was lower in PCOS-with-SCH than in PCOS and control patients indicating mild thyroid dysfunction at a statistical but not clinical level. Regarding endocrine profiles between PCOS and PCOS-with-SCH, the discrepant results suggest heterogeneity among studies precluding unbiased comparisons.

## Conclusion

The current study demonstrated that anthropometrical parameters and ovarian morphology were similar in PCOS and PCOS-with-SCH patients. Signs of hyperandrogenism were present in both groups. Regarding hormones, only C-peptide was higher in PCOS group. TSH correlated with total cholesterol, insulin, and prolactin. Collectively, current data indicated that the exclusion criterion “thyroid dysfunction” should be standardized before PCOS diagnosis. Subclinical hypothyroidism as manifested by a raised TSH with normal free T4, should not be used as a exclusion criterion for a diagnosis of PCOS. Studies designed to treat PCOS-with-SCH with thyroxin before excluding them from true PCOS group are still needed.
